# Peripheral Tumor Location Predicts a Favorable Prognosis in Patients with Resected Small Cell Lung Cancer

**DOI:** 10.1155/2022/4183326

**Published:** 2022-11-25

**Authors:** Yina Gao, Yangyang Dong, Yingxu Zhou, Gongyan Chen, Xuan Hong, Qingyuan Zhang

**Affiliations:** Department of Medical Oncology, Harbin Medical University Cancer Hospital, Harbin, China

## Abstract

**Background:**

Small cell lung cancer (SCLC) is an aggressive malignancy. Surgical resection is currently only recommended for clinical stage I patients who have been carefully staged. The clinical outcomes of patients with resected SCLCs vary because the disease is highly heterogeneous, suggesting that selected patients could be considered for surgical resection depending on their clinical and/or molecular characteristics.

**Methods:**

We collected data on a retrospective cohort of 119 limited-stage SCLC patients who underwent lobectomy with mediastinal lymph node dissection from March 2013 to March 2020 at Harbin Medical University Cancer Hospital. Correlations were derived using Fisher's exact test. Models of 2-year and 3-year survival were evaluated by deriving the area under receiver operating characteristic curves. Kaplan–Meier and Cox regression analyses were used to evaluate significant differences between the survival curves and hazard ratios.

**Results:**

The median disease-free survival (DFS) was 35.9 months (range 0.9–105.3 months), and the median overall survival (OS) was 45.2 months (range 4.8–105.3 months). Univariate analysis showed that TNM stage was significantly correlated with DFS and OS. The 2-year disease-free rates of patients with stage I, II, and III disease were 76.4%, 50.5%, and 36.1%, respectively, and the 3-year OS rates were 75.9%, 57.7%, and 34.4%, respectively. In pN + patients, multiple (or multiple-station) lymph node involvement significantly increased recurrence and reduced survival compared with patients with single or single-station metastases. Patients with peripheral SCLCs evidenced significantly better DFS and OS than did patients with central tumors. Multivariate analysis showed that TNM stage and tumor location were independently prognostic in Chinese patients with resected limited-stage SCLC. A combination of TNM stage and tumor location was helpful for prognosis.

**Conclusions:**

TNM stage and tumor location were independently prognostic in Chinese patients with resected SCLCs. Patient stratification by tumor location should inform the therapeutic strategy. The role of surgical resection for limited-stage SCLC patients must be reevaluated, as this may be appropriate for some patients.

## 1. Introduction

Lung cancer is the most common cancer worldwide. In 2020, it was the leading cause of cancer-related death in males and the second most common cause in females [1]. Lung cancer includes both nonsmall cell lung cancer (NSCLC) and small cell lung cancer (SCLC), which differ pathologically. SCLC is an aggressive malignancy characterized by rapid growth, early metastasis, and poor prognosis. Although the proportion of SCLCs among all histological lung cancer types has decreased markedly in the United States, significant increases in the proportions of both population- and hospital-based SCLC cases have been noted in some Chinese regions in recent years, especially among the elderly [2, 3]. After appropriate treatment, about 20% of patients with limited-stage (LS) SCLC (as defined by the Veterans Administration Lung Study Group) survive. Unfortunately, for the other 80%, and for all patients with extensive-stage (ES) disease, the outcomes remain poor, despite transitory responses to initial chemotherapy and radiotherapy [4]. Although the standard front-line treatment for ES SCLC changed when two randomized phase 3 trials revealed improved survival with the addition of atezolizumab or durvalumab to platinum plus etoposide [5, 6], survival remains modest compared with that of patients with other solid tumors (e.g., NSCLC). For most LS SCLC patients, concurrent chemoradiation is the standard of care; only patients with stage I to stage IIA (T1-2, N0) SCLC are recommended for surgery. Thus, fewer than 5% of SCLC patients undergo surgery, and most SCLC patients are diagnosed (unexpectedly) with SCLC during surgery [7, 8]. Some evidence supports a role for surgery when LS SCLC patients have been precisely staged. It has been suggested that lobectomy for selected LS SCLC patients improved local control and survival [9–13]. Stage is the major prognostic factor for resected SCLC patients; however, individual survival times differ even among patients of the same stage. Apart from TNM staging, several studies have reported that certain clinical characteristics/indicators (sex, age, tumor location, adjuvant chemotherapy status, and lymph node metastatic ratio) correlated with the clinical outcomes of resected SCLC patients [9, 14]. However, most studies evaluated the cohort from the Surveillance, Epidemiology, and End Results (SEER) database; clinical information was thus limited. Recent retrospective works indicated that two indices of inflammation, the neutrophil-to-lymphocyte ratio (NLR) and advanced lung cancer inflammation index (ALI), were prognostic in resected SCLC patients [15, 16]. Thus, we here combined known and new prognostic indicators of a retrospective cohort. The results from this study improved our understanding of how baseline patient and tumor characteristics affect the prognosis of Chinese patients with resected LS SCLCs.

## 2. Patients and Methods

We retrospectively studied a cohort of 119 LS SCLC patients who underwent lobectomy with mediastinal lymph node dissection from March 2013 to March 2020 at Harbin Medical University Cancer Hospital. We enrolled all patients with SCLC only (as confirmed histopathological analysis). This study was approved by the Medical Ethics Committee of Harbin Medical University Cancer Hospital (Harbin, China). The need for informed patient consent was waived because of the retrospective nature of the study. The primary endpoints were disease-free survival (DFS) and overall survival (OS). DFS was defined as the time from surgery to disease recurrence or death, and OS was defined as the time from surgery to death from any cause, or to May 2022 for patients who remained alive. Clinicopathological and treatment characteristics, including sex, age (<65 or ≥65 years), smoking history (never-smokers versus current smokers), pathological TNM stage, tumor location, number of lymph node metastases, the neoadjuvant or/and adjuvant therapy regimen (chemotherapy or radiotherapy), and preoperative hematological parameters (e.g., the neutrophil, lymphocyte, and platelet counts), were abstracted from the electronic medical record system. Current smokers included those with a smoking index ˃400 packs per year throughout life and those who had quit for ˂5 years; the remaining patients were defined as never-smokers. Staging after surgery employed the 8^th^ edition of the American Joint Committee on Cancer criteria [17]. Primary tumor location was defined as central or peripheral upon bronchoscopy performed by experienced specialists. Central tumors involved the segmental or more proximal bronchi. Tumors with primary sites distal to the subsegmental bronchi and tumors that were not evident via bronchoscopy were defined as peripheral SCLCs. The NLR was calculated by dividing the absolute neutrophil count by the absolute lymphocyte count, and the PLR was the platelet count divided by the absolute lymphocyte count.

All statistical analyses were performed with the aid of GraphPad Prism version 8 (Prism Software Inc., San Diego, CA, USA) and SPSS version 26 (SPSS Software Inc., Chicago, IL, USA). Numerical variables were presented as the mean ± standard deviation, otherwise presented as the median and range. One-way ANOVA analyses were used for the analysis of the NLR and PLR in patients with different stages. Models of the NLR and PLR at 2-year DFS and 3-year OS were evaluated using the area under the receiver operator characteristic curves (AUCs). Correlations were derived using the Pearson chi-squared test or the Fisher exact test to compare categorical variables. Kaplan–Meier analysis and the log-rank Mantel–Cox test were used to compare survival curves and hazard ratios (HRs). To identify independent prognostic factors, a multivariate survival analysis with HRs proceeded using Cox's regression model of factors that were significant on univariate analysis. All *P* values were two-sided, and *P* < 0.05 was considered to indicate statistical significance.

## 3. Results

### 3.1. Clinical and Treatment Characteristics

The characteristics of the 119 patients are summarized in Table 1. In total, 62 (52.1%) experienced disease recurrence or died. The median time to recurrence was 11.2 months (range 0.9–70.2 months). The median DFS was 35.9 months (range 0.9–105.3 months), and the disease-free rates at 1, 2, and 3 years were 71.4%, 58.8%, and 50.4%, respectively. Of 112 patients for whom survival outcomes were available, 52 died (46.4%). The median OS was 45.2 months (range 4.8–105.3 months), and the 2-, 3-, 4-, and 5-year survival rates were 75.0%, 59.8%, 46.2%, and 33.9%, respectively. The median age of all patients was 56 years (range 35–77 years) and 63.9% were male; 63.9% had smoked more than 20 pack years of cigarettes. The proportions with stage I, II, and III disease after surgery were 46.2%, 23.5%, and 30.3%, respectively. Of 119 patients, 102 received adjuvant chemotherapy and 28 received neoadjuvant chemotherapy before resection. Only eight patients received postoperative mediastinal radiotherapy and 10 received prophylactic cranial irradiation.

### 3.2. Prognostic Utilities of Clinical Characteristics and Clinicopathological Features for Resected LS SCLC Patients

**Figure 1:**
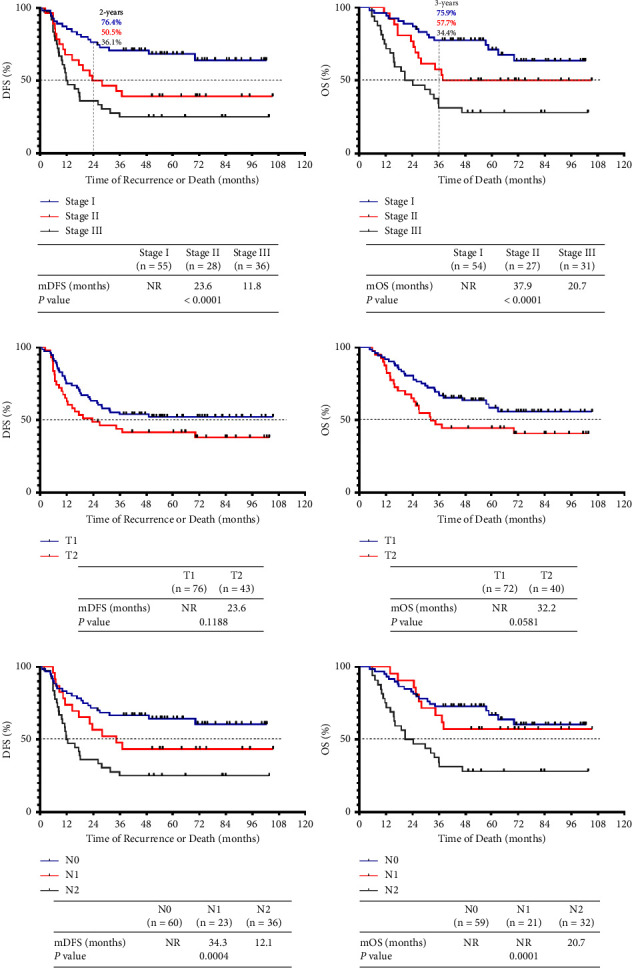
Identification and prognostic significance of TNM stage in LS SCLC-resected patients. (a) The DFS (*n* = 119) and OS (*n* = 112) curves of LS SCLC-resected patients by TNM stage (I, II, and III). (b) The DFS (*n* = 119) and OS (*n* = 112) curves of LS SCLC-resected patients by T stage (T1 and T2). (c) The DFS (*n* = 119) and OS (*n* = 112) curves of LS SCLC-resected patients by TNM stage (N0, N1, and N2).

Female sex, younger age, and stage I disease were previously shown to be associated with a better prognosis in LS SCLC patients [14]. Thus, we first evaluated the prognostic significance of baseline clinical characteristics (Table 2). Univariate analysis showed that TNM stage was significantly correlated with both DFS (*P* < 0.0001) and OS (*P* < 0.0001). The 2-year disease-free rates for patients with stage I, II, and III disease were 76.4%, 50.5%, and 36.1%, respectively, and the 3-year figures were 75.9%, 57.7%, and 34.4%, respectively (Figure 1(a)). Sex, age, or smoking status was not significantly prognostic, although females and never-smokers exhibited longer survival times. In addition, several pathological tumor characteristics, including the T and N stages, were prognostic. Two patients initially classified as T2 stage were pathologically confirmed to be T3 stage and were excluded for analyses of the correlation between T stage and prognosis due to a small sample size. The results showed that there was no statistical significance for the correlation between T stage and prognosis; however, it seems that patients with T2 stage trended toward a shorter DFS (23.6 months vs. NR, *P* = 0.1188) and OS (32.2 months vs. NR, *P* = 0.0581) than T1 stage (Figure 1(b)). Univariate analysis of pathological N stages in predicting prognosis revealed that N stages were significantly associated with DFS (*P* = 0.0004) and OS (*P* = 0.0001) in resected SCLC, which was consistent with previous studies [9, 14] (Figure 1(c)).

**Figure 2:**
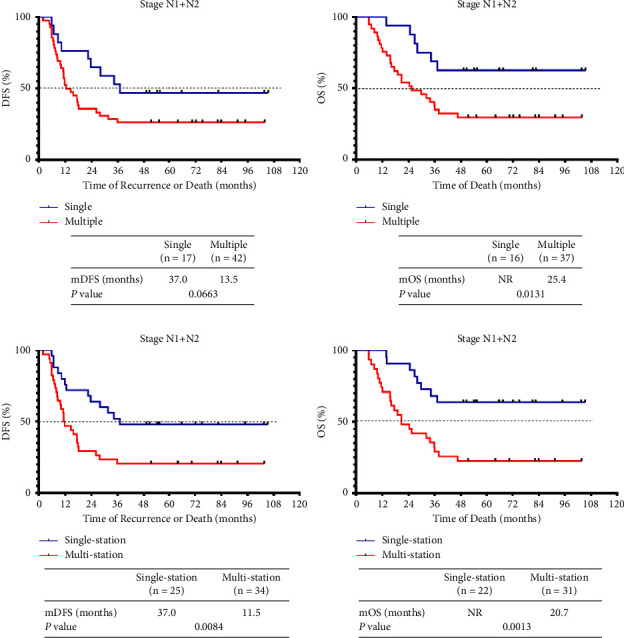
Prognostic utility of lymph node involvement for patients diagnosed with pN-positive SCLC after surgery. (a) The DFS (*n* = 59) and OS (*n* = 53) curves of stage pN1 and pN2 SCLC patients after surgery by lymph node involvement (single and multiple lymph node metastases). (b) The DFS (*n* = 59) and OS (*n* = 53) curves of stage pN1 and pN2 SCLC patients after surgery by lymph node station involvement (single- and multistation lymph node metastases).

**Figure 3:**
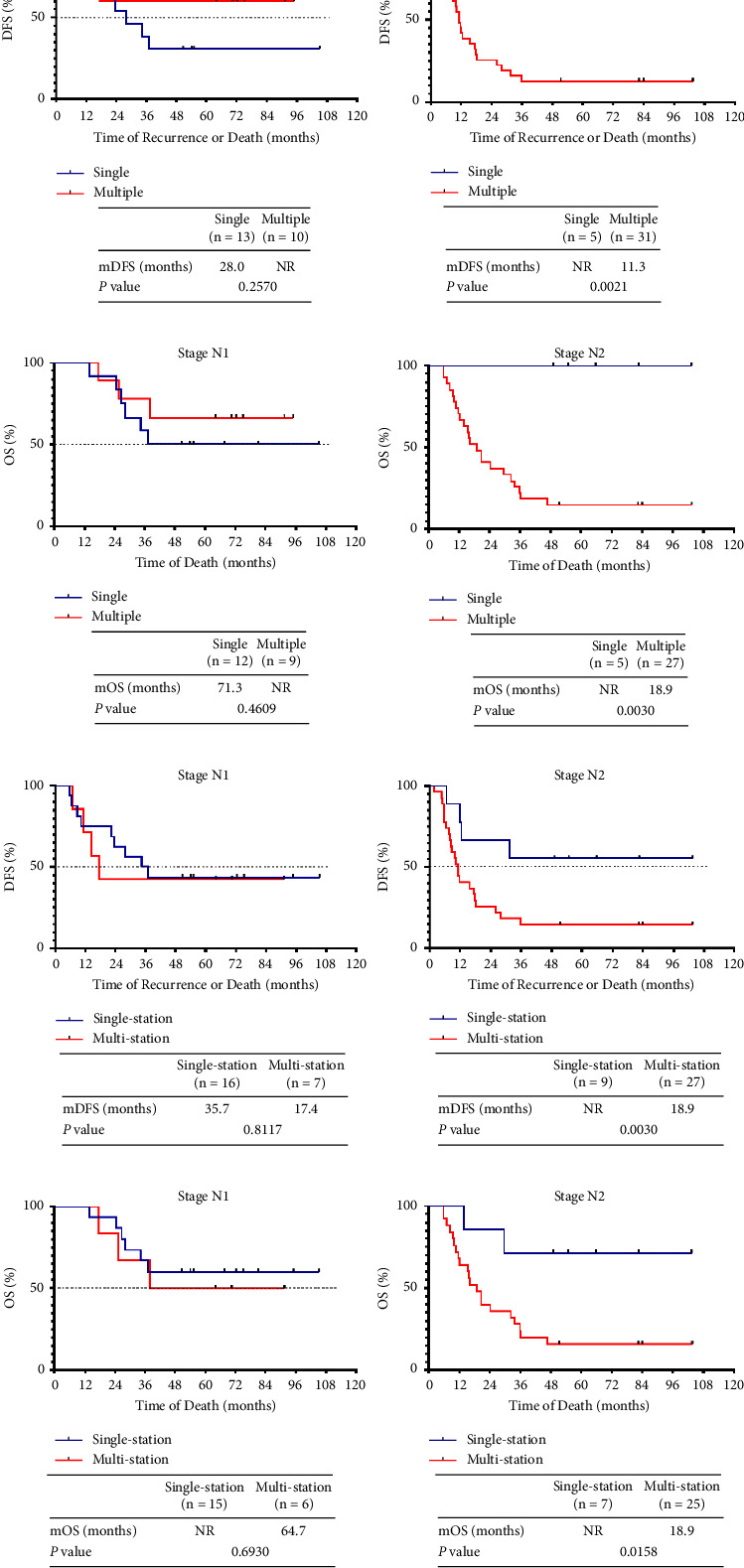
Prognostic utility of lymph node involvement by N stage in patients with resected LS SCLC. (a) The DFS curves of resected SCLC patients with stage pN1 (*n* = 23) and pN2 (*n* = 36) by the number of involved lymph nodes. (b) The survival curves of resected SCLC patients with stage pN1 (*n* = 23) and pN2 (*n* = 36) by the number of involved lymph nodes. (c) The DFS curves of resected SCLC patients with stage pN1 (*n* = 23) and pN2 (*n* = 36) by the numbers of involved lymph node stations. (d) The survival curves of resected SCLC patients with stage pN1 (*n* = 23) and pN2 (*n* = 36) by the number of involved lymph node stations.

Resected NSCLC patients with lymph node metastases varied in terms of prognosis, but multiple lymph node station metastases correlated with an inferior prognosis [18]. It was previously shown that the sites and numbers of metastatic lymph nodes correlated with the prognosis of resected N2 SCLC patients [19]. Thus, we analyzed the association between metastatic lymph node status and DFS and OS in pathological stage N+ patients. Those with several metastatic lymph nodes had recurrence sooner than did patients with single metastatic lymph nodes (13.5 vs. 37.0 months, *P* = 0.0663), and the survival time of the former patients was significantly shorter (25.4 months vs. NR, *P* = 0.0131) (Figure 2(a)). LS SCLC patients with multiple-station lymph node metastases exhibited a shorter time to recurrence (11.5 vs. 37.0 months, *P* = 0.0084) and poorer survival than did patients with single-station metastatic lymph nodes (20.7 months vs. NR, *P* = 0.0013) (Figure 2(b)). Further analysis stratified by N stage revealed that this was true only for patients with N2-stage disease (Figure 3). Such patients with metastases in multiple lymph nodes of several stations exhibited much earlier relapses and death as compared with those with single affected lymph nodes.

### 3.3. Impact of Primary Tumor Location on Prognosis

**Figure 4:**
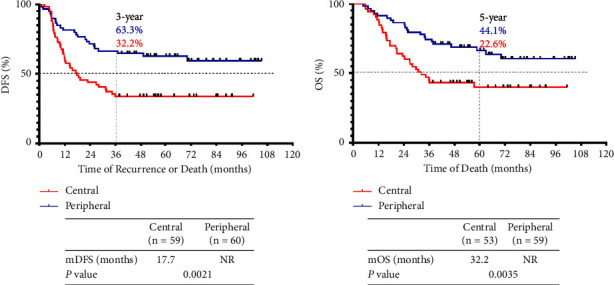
Identification and prognostic significance of tumor location in resected LS SCLC patients. (a) The DFS curves of resected LS SCLC patients with central (*n* = 59) and peripheral (*n* = 60) tumors. (b) The survival curves of resected LS SCLC patients with central (*n* = 59) and peripheral (*n* = 60) tumors.

SCLC typically presents as a large hilar mass accompanied by bulky mediastinal lymphadenopathy. Most patients who were (unexpectedly) diagnosed with pathological SCLC presented with solitary peripheral nodules before surgery. Two prior studies reported that peripheral SCLCs more commonly expressed TTF-1 than did central tumors and were associated with a poorer prognosis [20, 21]. However, the clinical significance of tumor origin in resected LS SCLC patients remains unclear. Out of all our patients, 60 presented with peripheral tumors (53.3% stage I (32/60) and 46.7% stages II and III (28/60)) and 59 with central tumors (39.0% stage I (23/59) and 61.0% stages II and III (36/59)). The TNM stages of our peripheral and central SCLC patients did not differ (*P* = 0.142). TTF-1 immunoreactivity was apparent in 87 tumors, and peripheral tumors expressed TTF-1 more frequently (91.7%, 44/48) than did central SCLCs (71.8%, 28/39) (*P* = 0.0215). Unlike previous studies of unresected SCLC patients, we found that patients with peripheral SCLCs exhibited significantly longer DFS (*P* = 0.0021) and OS (*P* = 0.0035) than did those with central tumors (Figure 4). The median DFS and OS were 17.7 and 32.2 months, respectively, in those with central tumors compared with “not attained” in patients with peripheral tumors. The 3-year DFS and the 5-year OS were 32.2% and 22.6%, respectively, in those with central tumors, but 63.3% and 44.1%, respectively, in patients with peripheral tumors.

### 3.4. The Impacts of Inflammation-Based Scores on DFS and OS

**Figure 5:**
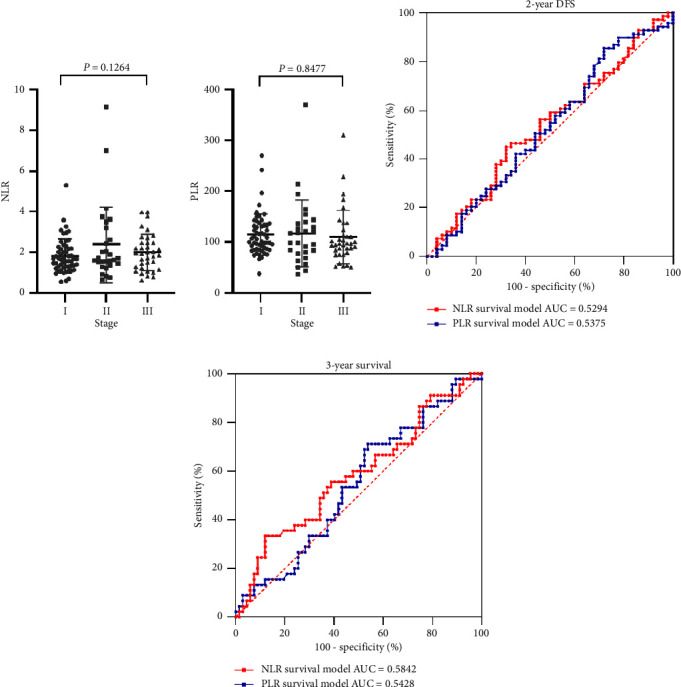
Correlations between the preoperative NLR, PLR, and TNM stage, and the prognosis of resected SCLC patients. (a) The preoperative NLRs of resected LS SCLC patients by the TNM stage. (b) The preoperative PLRs of resected LS SCLC patients by the TNM stage. (c) The 2-year DFS predictions of the receiver operator curves for NLR and PLR (*n* = 119). (d) The 3-year OS predictions of the receiver operator curves for the NLR and PLR (*n* = 112).

Previous studies have indicated that several hematological variables, including the lymphocyte count and NLR (both of which reflect immune system and inflammation status), correlated with LS SCLC prognosis [22]. A study of Caucasian patients with stage I or II resected SCLC showed that the preoperative NLR (but not the PLR) was associated with longer OS [16]. We first sought to assess the correlations between the NLR and PLR and TNM stage. The median NLRs and PLRs of all patients were 1.711 (0.546–9.160) and 100.0 (36.36–370.40), respectively. The median NLRs in patients with stage I, II, and III disease were 1.817 ± 0.839, 2.379 ± 1.885, and 1.999 ± 0.891, respectively, and the median PLRs in patients with stage I, II, and III disease were 114.4 ± 40.84, 117.2 ± 65.95, and 110.1 ± 52.92, respectively. No significant correlation between the NLR or PLR and tumor stage was apparent (Figures 5(a) and 5(b)). The AUCs for DFS at 2 years and OS at 3 years revealed that the NLR and PLR failed to predict prognosis (Figures 5(c) and 5(d)).

### 3.5. Multivariable Cox's Proportional Hazard Analysis of Prognosis

To identify the independent predictors for prognosis, we performed multivariate Cox's regression analyses of DFS and OS. Variables identified as risk factors in the prior studies as well as our results in univariate analysis were enrolled, including sex, age, smoking status, TNM stage, T stage, N stage, and tumor location. Given the impact of therapy after surgery on prognosis, several adjuvant treatments, including adjuvant chemotherapy, radiotherapy, and PCI, were added to the variables. The multivariate analysis revealed that TNM stage and tumor location were independently prognostic of DFS and OS after adjustment for confounders (Table 3).

**Figure 6:**
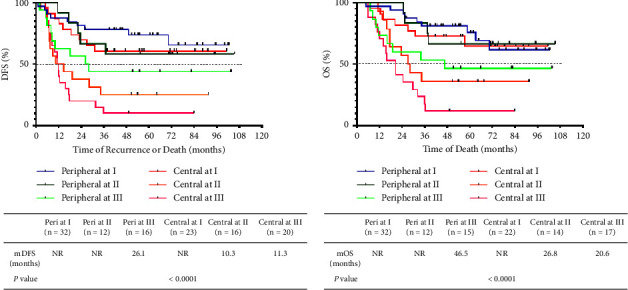
Kaplan–Meier curves of the DFS and OS of resected LS SCLC patients by TNM stage combined with tumor location. (a) The DFS curves of resected LS SCLC patients by TNM stage (I, II, and III) and tumor location (central and peripheral) (six groups). (b) The OS curves of resected LS SCLC patients by TNM stage (I, II, and III) and tumor location (central and peripheral) (six groups).

Considering TNM stage and tumor location as independent predictive factors, we next integrated these two factors to predict prognosis. Survival analysis showed that patients with stage I or II peripheral tumors and stage I central tumors enjoyed better prognoses than those with stage III peripheral tumors and stage II or III central tumors (Figure 6). Thus, integration of TNM stage and tumor location was meaningfully prognostic in terms of both DFS and OS.

## 4. Discussion

SCLC is aggressive, and surgical resection is currently recommended (only) for clinical stage I patients who have been carefully staged. In practice, most SCLC patients who undergo surgical resection are diagnosed intraoperatively or postoperatively. The largest published retrospective analysis of the SEER database found that patients with stage I or II SCLC undergoing resection had better survival than did nonsurgical patients (median OS 34.0 versus 16.0 months) [23]. Similar retrospective studies, including patients with LS SCLC, also suggested that the role of surgery as an LS SCLC treatment required reevaluation [24, 25]. The clinical outcomes of resected SCLC patients of the same stage vary, as the disease is heterogeneous. This suggests that certain patients might benefit from surgical resection. However, the clinical and/or molecular characteristics that might identify such patients are unknown. To the best of our knowledge, this is the first attempt to employ tumor location to predict the DFS and OS of resected LS SCLC patients. We found that both tumor location and TNM stage were independently prognostic.

Central and peripheral SCLCs differ in terms of genome (in) stability, driver gene somatic copy numbers, and mutational signatures, suggesting that the biological characteristics differ [26]. Previous studies regarding the significance of SCLC tumor location have yielded inconsistent results, and another study showed that peripheral SCLCs expressed TTF-1 more frequently than did central SCLCs and were associated with a poorer prognosis [20]. We agree (only) in terms of the TTF-1 expression frequency. We found the exact opposite in terms of prognosis. The reason for this difference could be that the study populations differed. Nevertheless, a similar study enrolling a larger population found that peripheral SCLC was associated with longer OS than that of central SCLC [27], which was consistent with our study. Notably, our focus was on resected LS SCLC patients. The populations in the studies cited above were dominated by ES SCLC patients receiving systematic therapy.

Our survival analyses showed that stage II peripheral SCLC patients after surgery experienced a prognosis similar to that of central SCLC patients of stage I, for whom surgical resection is currently recommended, suggesting that selected patients with N1 SCLC might benefit from surgery. Given the inferior DFS and OS of peripheral SCLC patients of stage III and central SCLC patients of stages II and III, our study supports the idea that such patients should receive the systematic therapy of the current guidelines.

Previous studies of NSCLC patients who underwent surgery reported that the numbers and sites of involved lymph nodes correlated significantly with prognosis, especially for N2-stage patients [28–30]. We describe here, for the first time, how lymph node involvement affects the prognosis of pN1- and N2-stage resected SCLC patients. Patients with multiple involved lymph node numbers/stations experienced a median OS similar to that of patients receiving chemoradiation, indicating the importance of preoperative staging. Stratified analysis by N stage revealed that lymph node status was prognostic principally in patients of pN2 status, further emphasizing the high-level heterogeneity of pN2-stage resected SCLC, consistent with the findings of a prior study of N2-stage resected SCLC patients [31]. When we compared survival with the N stage, we found that N1-stage patient survival was similar to that of N0-stage patients and significantly longer than that of N2-stage patients, suggesting that some patients with N1-stage SCLC (as revealed by preoperative imaging) should be considered for surgical resection. Similarly, a previous study of stage I and stage II SCLC patients in the SEER database supported possible surgical resection to treat stage II SCLC [32].

This study has some limitations. This was a single-center retrospective study, and we were thus unable to consider all relevant factors. In addition, selection bias may have been in play, contributing to differences between our findings and those of others. A recent study reported that the preoperative NLRs and PLRs predicted the survival of patients with LS SCLC; however, we did not find this [33]. However, the baseline characteristics of patients differed between the two studies. We enrolled more patients with stage I (46.2%) than did the cited work (22.3%). Our median NLR and PLR values were 1.711 and 100.0, respectively, which are lower than those in the cited study. Zeng et al. [34] enrolled some patients with histologically combined SCLC, while we enrolled only pure SCLC patients. However, on univariate analysis, we found that females and never-smokers exhibited longer survivals, as have previous studies. Our small sample size and short follow-up period may limit the statistical power of the analyses of correlations between sex and smoking and prognosis.

## 5. Conclusion

We found that TNM stage and tumor location were independently prognostic in Chinese patients with resected LS SCLC. This aids the evaluation of how surgery affects the prognoses of different groups and suggests that patient stratification by tumor location would optimize therapies. We found that the survival of pN1- and pN0-stage patients was similar and significantly longer than that of pN2 patients. In addition, the lymph node involvement status of pN2-stage resected SCLC patients was meaningful in terms of DFS and OS prognoses. Future prospective studies should reevaluate the utility of surgical resection in selected LS SCLC patients with lymph node metastases.

## Figures and Tables

**Table 1:** Clinicopathological and treatment characteristics.

Characteristics	Total (*n* = 119)
Age	
Median (years)	56 (35–77)
<65	95 (79.8%)
≥65	24 (20.2%)
Sex	
Female	43 (36.1%)
Male	76 (63.9%)
Smoking	
Never-smoker	43 (36.1%)
Current smoker	76 (63.9%)
Staging	
IA	43 (36.1%)
IB	12 (10.1%)
IIA	4 (3.4%)
IIB	24 (20.2%)
IIIA	35 (29.4%)
IIIB	1 (0.8%)
Treatment	
Adjuvant chemotherapy	102 (85.7%)
Neoadjuvant chemotherapy	28 (23.5%)
Postoperative mediastinal RT	8 (6.7%)
PCI	10 (8.4%)

**Table 2:** Patient distribution and characteristics associated with DFS and OS.

Variable	DFS			OS		
	Number (%)	Medium (months), HR (95% CI)	*P* value	Number (%)	Medium (months), HR (95% CI)	*P* value
Gender			0.3077			0.0915
Male	76 (63.9)	31.6, 1.318 (0.790–2.197)		71 (63.4)	57.3, 1.666 (0.957–2.899)	
Female	43 (36.1)	70.2, 0.759 (0.455–1.266)		41 (36.6)	NR, 0.600 (0.345–1.045)	
Age (years)			0.6729			0.9895
<65	95 (79.8)	35.9, 1.110 (0.688–1.791)		89 (79.5)	NR, 1.004 (0.517–1.953)	
≥65	24 (20.2)	73.3, 0.901 (0.558–1.453)		23 (20.5)	58.6, 0.996 (0.512–1.935)	
Smoking status			0.2678			0.0616
Never-smokers	43 (36.1)	NR, 0.741 (0.445–1.234)		39 (34.8)	NR, 0.583 (0.334–1.019)	
Current smokers	76 (63.9)	28.0, 1.349 (0.810–2.247)		73 (65.2)	63, 1.715 (0.982–2.997)	
TNM stage			<0.0001			<0.0001
I	55 (46.2)	NR, 0.444 (0.215–0.918)		54 (48.2)	NR, 0.528 (0.239–1.167)	
II	28 (23.5)	23.6, 0.649 (0.360–1.172)		26 (23.2)	37.9, 0.500 (0.260–0.963)	
III	36 (30.3)	11.8, 3.388 (1.798–6.384)		32 (28.6)	20.7, 3.643 (1.815–7.313)	
T stage			0.1188			0.0581
T1	76 (63.9)	NR, 0.672 (0.395–1.142)		72 (64.3)	NR, 0.568 (0.317–1.020)	
T2	43 (36.1)	23.6, 1.489 (0.876–2.531)		40 (35.7)	32.2, 1.759 (0.981–3.157)	
N stage			0.0004			0.0001
N0	60 (50.4)	NR, 0.571 (0.269–1.212)		59 (52.7)	NR, 0.808 (0.355–1.837)	
N1	23 (19.3)	34.3, 0.551 (0.296–1.026)		21 (18.7)	NR, 0.395 (0.198–0.790)	
N2	36 (30.3)	12.1, 2.871 (1.558–5.292)		32 (28.6)	20.7, 3.083 (1.575–6.037)	

NR, not reached.

**Table 3:** Multivariate analysis for DFS and OS in resected LS SCLC.

Variable	DFS			OS		
	HR	95% CI	*P* value	HR	95% CI	*P* value
Gender (female vs. male)	0.793	0.450–1.398	0.422	0.615	0.323–1.172	0.140
Age (≥ 65 y vs. <65 y)	1.179	0.601–2.314	0.632	1.175	0.580–2.381	0.655
Smoking status (current vs. never)	1.138	0.627–2.066	0.672	1.330	0.689–2.568	0.396
TNM stage (III vs. II vs. I)	4.310	1.317–14.101	0.016	3.892	1.160–13.062	0.028
T stage (T2 vs. T1)	0.981	0.561–1.717	0.947	1.050	0.572–1.930	0.874
N stage (N2 vs. N1 vs. N0)	0.419	0.137–1.274	0.125	0.490	0.159–1.510	0.214
Tumor location (peripheral vs. central)	0.471	0.276–0.804	0.006	0.495	0.276–0.888	0.018
Adjuvant chemo (yes vs. no)	1.216	0.670–2.206	0.520	1.133	0.604–2.125	0.697
Adjuvant radio (yes vs. no)	1.140	0.413–3.146	0.800	1.035	0.334–3.204	0.953
PCI (yes vs. no)	0.746	0.220–2.533	0.639	0.385	0.050–2.940	0.357

## Data Availability

The data used to support the findings of this study are available from the corresponding authors upon request.
